# The Validity and Reliability of the Polish Version of the PedsQL™ Gastrointestinal Symptoms Module for Pediatric Patients (Aged 2–18)

**DOI:** 10.3390/jcm14041227

**Published:** 2025-02-13

**Authors:** Julia Leszkowicz, Małgorzata Kasprowicz-Janisz, Anna Kotarska, Wojciech Nazar, Magdalena Dettlaff-Dunowska, Justyna Napora, Tomasz Mazurek, Katarzyna Plata-Nazar, Agnieszka Szlagatys-Sidorkiewicz

**Affiliations:** 1 Department of Paediatrics, Gastroenterology, Allergology and Nutrition, Medical University of Gdańsk, Nowe Ogrody 1-6, 80-803 Gdańsk, Poland; 2 Linguistic Validation Consultant at the Mapi Research Institute, Division of English Language Translation Studies, Institute of English and American Studies, University of Gdansk, Wita Stwosza 51, 80-308 Gdańsk, Poland; 3 Polish Society for Health Programs, Polskie Towarzystwo Programów Zdrowotnych, Dębowa 30, 80-204 Gdańsk, Poland; 4 Laboratory of Experimental and Translational Allergology, Division of Allergology Department of Pulmonology & Allergology, Medical University of Gdańsk, Mariana Smoluchowskiego 17, 80-214 Gdańsk, Poland; 5 Department of Orthopaedics and Traumatology, Faculty of Medicine, Medical University of Gdańsk, Nowe Ogrody 1-6, 80-803 Gdańsk, Poland

**Keywords:** health-related quality of life, linguistic validity, reliability, gastroenterology, pediatrics, medical translation

## Abstract

**Background/Objectives:** Health-related quality of life has come to the forefront of the process of treatment. As a non-quantitative value, it requires the use of specialized tools to be measured. Despite the availability of general HRQoL assessment tools in Polish, a specific instrument for children with gastrointestinal (GI) disorders has not been developed. This study aims to describe the linguistic validity and reliability of the Polish version of the PedsQL™ Gastrointestinal Symptoms Module, which measured health-related quality of life (HRQoL) in children with gastrointestinal (GI) disorders. **Methods:** The PedsQL™ module, originally in English, was translated according to a structured process. Professional medical translators conducted the translations, and cognitive debriefing was performed with 35 respondents. Field testing involved 371 completed questionnaires (203 adults and 168 children). **Results:** Internal consistency was assessed using Cronbach’s alpha, with values generally exceeding 0.8 indicating high reliability. **Conclusions:** The final Polish version of the PedsQL™ GI Symptoms Module exhibits strong linguistic and content validity, making it suitable for both clinical trials and routine practice. Its use enables a comprehensive, culturally sensitive assessment of HRQoL in pediatric patients with GI disorders, thereby supporting individualized patient care and enhancing the physician–patient communication that is essential for effective treatment.

## 1. Introduction

Health-related quality of life (HRQoL) has a growing impact on both patients and physicians’ work [1], especially in gastroenterology, where a wide range of diseases originate in functional disorders. Those disorders are strictly connected to well-being [2]. Quality of life is a complex component of health assessments [3]. Health-related quality of life itself can be defined as a multidimensional concept that describes the physical, social, and psychological aspects of well-being and functioning. Furthermore, HRQoL can include both objective and subjective perspectives in each domain [4]. It enables the evaluation of different, non-quantitative domains assessing a child’s functioning and the impact on their everyday life [1].

Only a few generic questionnaires designed for children and written in Polish exist that have been validated for the assessment of HRQoL. European Kidscreen-52 is one of the tools used to evaluate HRQoL among the Polish population [5], but it can also be used for specific diseases [6]. Other disease-centered tools, such as EA-QOL [7] for esophageal atresia or PAQLQ for asthma [8], are available in Polish. The PedsQL 4.0 Generic Core Scales and Module [9] was applied in some Polish studies concerning HRQoL in general [10,11,12], inter alia, but not gastroenterological problems specifically.

Such a complex questionnaire that can assess the quality of life of patients with gastrointestinal disorders has yet to be created or translated to Polish. The authors of this paper observed the need to adapt a tool that can systematically evaluate the problems of children with GI tract disorders in their local population. The PedsQL™ Gastrointestinal Symptoms Module presents a variety of customized questionnaires for different age groups and disorders and is a reliable and valid tool for measuring HRQoL in children with different GI disorders [13,14]. It also presents the dual perspective of the child and parent proxy. This study aims to describe the linguistic and content validity of the Polish version of the PedsQL™ Gastrointestinal Symptoms Module, which measures health-related quality of life (HRQOL) in children with gastrointestinal (GI) disorders.

## 2. Materials and Methods

### 2.1. Questionnaire Design

The PedsQL™ Gastrointestinal Symptoms Module was originally developed by Dr. James Varni in American English. The questionnaire comprises 74 items divided into 14 domains and measures the gastrointestinal-related quality of life in pediatric patients. Translations were required to provide high-quality and semantic consistency with the original version [15].

Before starting the project, permission was obtained from the Mapi Research Institute and author Dr. James W. Varni to translate the questionnaires from US English to Polish and validate them according to their study protocol [16].

In the original English version, 7 separate age groups were distinguished: from 2 to 4 (only parent proxy reports), from 5 to 7, from 8 to 12, and from 13 to 18 years of age (both child and parent proxy reports) with functional and organic GI disorders. The grading scale consisted of 5 answers—never, almost never, sometimes, often, and almost always—with the exception of the proxy reports from the age group involving young children (5 to 7 years) that consisted of 3 answers—not at all, sometimes, and a lot—and it also included icons of smiley faces.

### 2.2. Linguistic Validity

**Figure 1:**
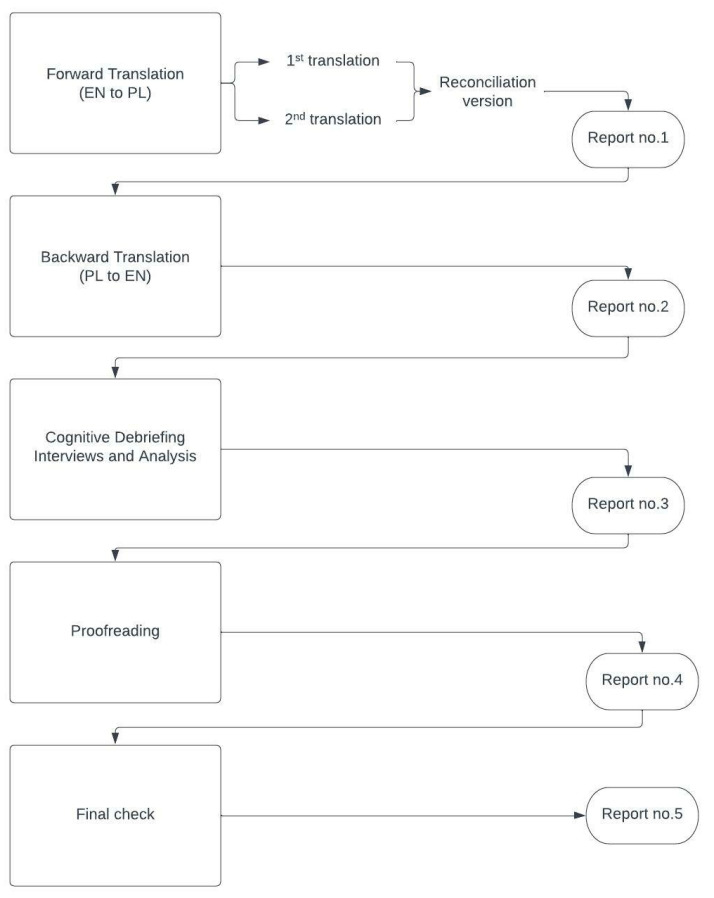
The stages of the validation process.

The whole procedure of translation and validation was comprehensively described by the Mapi Research Institute [16]. The protocol consists of five steps. Each is accompanied by a report. Figure 1 shows the steps used in the process.

The first step consisted of a forward translation of the original version from US English to Polish, performed by two independent professional translators both born and raised in Poland, with a background in medical language, for whom Polish is their mother tongue. Following the translation stage, a linguistic specialist performed a reconciliation step and drafted one conceptually equivalent version of each of the papers dedicated to individual age groups. Back translation was performed by a native speaker of English who also knows Polish. After this step, the cognitive debriefing stage was carried out. A total of 35 respondents were recruited, providing 7 groups with 5 respondents each. All children were patients of the Clinical Ward of Paediatrics, Gastroenterology, Allergology and Paediatric Nutrition in Gdansk, recruited from the ward or outpatient clinic. All adults were parents of the patients hospitalized at the same ward or outpatient clinic.

The interviews were conducted between 21 May 2022 and 13 July 2022 by a professional medical translator with the help of physicians working at the pediatric ward and took place onsite. Each participant was given sufficient time to fill in the questionnaire. Firstly, every respondent was asked for their consent to record the interview. Then, the respondents were asked whether they understood every sentence of the questionnaire and were given an explanation of any problematic words. Finally, each recording was analyzed by the project manager, and necessary amendments were made in the translations. The final report was approved by the Mapi Trust Institute and Dr. Varni, and the final version was produced.

### 2.3. Field Testing

After creating the final Polish version of the questionnaire, the next step was to verify the internal consistency of the questionnaire. A total of 203 (54.7%) complete questionnaires for adults and 168 (45.3%) for children were collected. The data were collected between 2 February 2023 and 14 July 2024. Only fully completed questionnaires were included in the analysis to ensure no data were missing from the study results. The data were analyzed following the official guidelines of the PedsQL 3.0 questionnaire. These scores were converted to a scale ranging from 0 to 100 based on the following rules: 0 to 100, 1 to 75, 2 to 50, 3 to 25, and 4 to 0. Higher scores indicated less severe gastrointestinal symptoms. The transformed scores from the questionnaires were treated as continuous variables.

According to the original questionnaire guidelines, the questions were grouped into 14 categories. The mean score for each category was calculated, and an overall average score from all individual questions was also computed. Cronbach’s alpha values were calculated to assess the internal consistency and reliability of the questionnaires. This was performed for each of the 14 categories and for all of the questions combined. A high alpha (close to 1) indicates that the items within the scale are highly correlated and measure the same underlying construct.

### 2.4. Statistical Analysis

Categorical data were summarized using counts, frequencies, and proportions. Statistical significance was determined by organizing the data into contingency tables and applying the Chi-square test with Yates’ correction. Continuous data, including the questionnaire scores, were described using means and 95% confidence intervals (95% CIs). The Shapiro–Wilk test was used to assess the normality of the data. Appropriate (Mann–Whitney U test or Kruskal–Wallis test) tests were used to identify statistically significant differences.

All analyses were performed using Python 3.10 with the Pandas 2.1.3 and Scikit-learn 1.2.1 libraries. A two-sided *p*-value of less than 0.05 (5%) was considered statistically significant.

## 3. Results

### 3.1. General Characteristics

A total of 371 fully completed questionnaires were analyzed, including 203 from adult respondents and 168 from child participants. Among children, the gender distribution was balanced across all groups (about 50%/50% male/female ratio; *p* > 0.05). For responses related to the children’s parents, the majority were obtained from mothers (over 85%). Educational attainment among parents was consistent across groups: approximately 50% completed higher education, 30% completed secondary education, and 20% completed primary/vocational education (*p* > 0.05).

For children with gastrointestinal (GI) diseases (functional disorders such as abdominal pain, nausea, constipation, or diarrhea; inflammatory bowel disease; gastroesophageal reflux, dysphagia, gastritis, pancreatitis, or coeliac disease; or anorexia), 100 responses (59.5%) were collected compared to 68 responses (40.5%) from the control group (patients of the pediatric orthopedics ward without GI tract conditions, fractures, or congenital defects). The mean age in the GI group was 10.8 years (95% CI: 10.0–11.6), and it was 11.5 years in the control group (95% CI: 10.6–12.4), with no significant difference (*p* = 0.284). Children in the control group scored significantly higher across all 74 questions and 14 main categories than those with GI diseases (*p* < 0.05).

For the parents of children with GI diseases, 121 responses (61.6%) were collected, while the control group yielded 78 responses (38.4%). The mean age of the children was 9.2 years (95% CI: 8.4–10.1) in the GI group and 10.5 years (95% CI: 9.5–11.5) in the control group (*p* = 0.082). Similarly to the children’s responses, the children in the control group had significantly higher scores across all questionnaire items and categories compared to those with GI diseases (*p* < 0.05). The results of the sample comparisons are shown in Table 1.

### 3.2. Factor Analysis

In order to verify the factor structure of the questionnaire under development, a confirmatory factor analysis (CFA) was carried out in IBM AMOS 9 software following a preliminary check of the normality assumption for the test item distributions. According to Watkins [17], raw data values should not exceed an absolute value of 2 for skewness and 7 for kurtosis. Minor deviations in skewness were found for test items associated with the factors Trouble swallowing and Blood in bowel movement (Appendix A). However, right-skewed distributions for these factors were present across all groups. An attempt to normalize them by removing outlier observations using the Mahalanobis distance function did not improve the distributions and would have required eliminating one-third of the collected observations. Consequently, this deviation was deemed natural for both factors, and the normal distributions observed in the remaining twelve factors justified conducting a CFA using the maximum likelihood (ML) method on the full sample of 371 observations. As recommended by Dash and Paul [18], in the case of composite reliability, the PCFI is a better indicator compared to the classic CFI due to the good assessment of model economy. In addition, measures of fit were included as follows: χ^2^ test (*p* > 0.05); RMSEA < 0.10; and SRMR < 0.08. Table 2 shows the factor loadings of the obtained solution and the AVE (average variance explained) and CR (composite reliability) indices.

The conducted factor analysis demonstrated an acceptable model fit to the collected data: χ^2^(2531) = 7189.328; *p* < 0.001; χ^2^/*df* = 2.841; PCFI = 0.768; RMSEA = 0.071 (95% CI [0.069, 0.072], *p* < 0.001); SRMR = 0.080. The removal of test items with factor loadings r < 0.50 did not yield a significant improvement in model fit, indicating that item elimination did not enhance the model’s structural integrity. A further evaluation of the average variance extracted (AVE) and composite reliability (CR) indices confirmed that the majority of questionnaire items exhibit factor loadings explaining more than 50% of the variance, reinforcing the theoretical robustness of the instrument. The CR values exceeded the threshold of 0.70 across all constructs, supporting adequate internal consistency and construct reliability. Additionally, convergent and discriminant validity were assessed using the Fornell–Larcker criterion [19], which states that the square of the inter-factor covariance should not exceed the AVE of the respective construct. The results confirm sufficient construct divergence, indicating that the instrument demonstrates acceptable convergent and discriminant validity within the tested model structure (Table 3).

### 3.3. Comparison of Control and Exposed Groups

To confirm the criterion validity of the tested instrument, a series of statistical analyses were conducted using IBM SPSS Statistics 30. First, the assumption of normality in the distribution of variables within the observation samples was examined using the Shapiro–Wilk test (Appendix B). The results indicate that the assumption of normality was violated, necessitating the use of non-parametric statistical methods. Subsequently, a group comparison was conducted between the control and exposed groups within the child and parent samples using the Mann–Whitney U test (Table 4) to determine whether children in the exposed group scored significantly higher on the tested instrument. Following this, a correlation analysis was performed using Spearman’s *rho* coefficient to assess the concordance between the parent and child scores (Table 5). Finally, a discriminant analysis was conducted (Table 6) to evaluate the accuracy of classification into the control and exposed groups based on the questionnaire scores across the identified factors.

Given the number of statistical tests conducted (Table 4), a Bonferroni correction was applied to adjust the significance threshold (α) in order to control for a Type I error. Based on this adjustment, the revised significance threshold was calculated as α = 0.002, indicating that all results below this threshold can be considered statistically robust and unlikely to be due to random chance. An analysis of both the child and parent responses revealed that all tested factors exhibited statistically significant differences between the control and exposed groups, with higher scale scores consistently being observed in the exposed group. An examination of effect sizes using the *r* coefficient indicated that the most pronounced differences were observed in the stomach pain and hurt, gas and bloating, and constipation scales irrespective of whether the questionnaire was completed by children or parents. Conversely, the smallest effect size was consistently noted for the trouble swallowing scale, which may mean that these symptoms were observed less frequently in the study sample.

The results (Table 5) show a strong overall agreement between the children’s self-reports and parents’ assessments of gastrointestinal symptoms, suggesting that parents can reliably recognize their children’s experiences. The highest agreement was found for symptoms like constipation, stomach pain, nausea, and blood in bowel movements, indicating that these issues are more noticeable to parents. However, there was slightly lower agreement for symptoms such as trouble swallowing and medicine use, which may be harder for parents to observe. Emotional and communication-related factors also showed moderate agreement, suggesting that while parents are generally aware of their children’s symptoms, some aspects of their experience might be less visible. These findings highlight the importance of considering both child and parent perspectives for a more complete understanding of gastrointestinal health.

The discriminant analysis (Table 6) demonstrates that the tested questionnaire effectively differentiates between the control and exposed groups based on symptom scores, with strong classification accuracy for both children and parents. The model correctly classified 88.2% of children and 89.7% of parents in the control group, as well as 78.0% of children and 83.2% of parents in the criterion group, indicating a high level of precision in distinguishing between groups. The key variables contributing to this differentiation include stomach pain, communication difficulties, and heartburn, which were generally higher in the criterion group. Conversely, factors like trouble swallowing and worry about bowel movements showed weaker differentiation. Overall, these results suggest that the tool provides a reliable measure for distinguishing individuals based on gastrointestinal health status, with the parents’ assessments slightly outperforming the children’s in classification accuracy.

### 3.4. Internal Consistency

The Cronbach’s alpha values (Table 7) in the children’s group were high for both the exposed and control groups, indicating strong internal consistency; the GI group had an alpha of 0.946 (95% CI: 0.940–0.952), while the control group’s alpha was 0.951 (95% CI: 0.947–0.955, *p* = 0.270). The Cronbach’s alpha values indicate strong reliability in both parent groups, with the GI group showing an alpha of 0.931 (95% CI: 0.925–0.937) and the control group showing an alpha of 0.953 (95% CI: 0.950–0.956, *p* < 0.001).

The value of 0.354 suggests less reliability in the control group. This variability might reflect changes in respondent interpretations or possible differences in sample characteristics between the two assessments. Moreover, subscales with few items (in the above case, two and four questions, respectively) usually give poor results compared to more copious subscales [20,21]. To minimize potential bias, it is recommended to provide respondents with additional explanations during data collection within those categories.

## 4. Discussion

Quality of life plays an increasingly important role in the treatment of diseases, especially functional and chronic ones. This study assessed the linguistic and psychometric properties of the Polish version of the PedsQL Gastrointestinal Module for pediatric patients (aged 2–18).

The validity analysis of the tested instrument confirms its effectiveness in distinguishing between control and exposed groups, as well as its reliability in capturing gastrointestinal symptoms across both child and parent reports. The Mann–Whitney U test demonstrated statistically significant differences between the groups, with higher symptom scores consistently being observed in the exposed group, particularly for stomach pain, bloating, and constipation. The strong correlations between child and parent responses indicate that parents can reliably assess their children’s symptoms, with the highest agreement being observed for constipation, stomach pain, and nausea, while symptoms like trouble swallowing and medication use showed slightly lower agreement. A discriminant analysis further validated the instrument’s classification accuracy, correctly assigning a high percentage of individuals to their respective groups based on symptom scores, with parental assessments slightly outperforming the children’s self-reports. Overall, these findings support the questionnaire’s criterion validity, convergent validity, and discriminant validity, affirming its suitability as a robust tool for evaluating gastrointestinal symptoms in children. The Cronbach’s alpha values for most items fall within a high range (generally above 0.7), indicating satisfactory internal consistency for the majority of the scales. The mean Cronbach’s alpha for all questions is high (0.946 and 0.951), indicating strong internal consistency for the questionnaire as a whole. Those results correspond with adaptations from other countries [20,22,23].

Following the comprehensive study protocol created by the Mapi Research Institute [24], it was possible to create a valid Polish version for surveying non-quantifiable aspects of health for use in academic research. The research was conducted by a small, qualified team, systematically, in conditions conducive to concentration and reflection on the questions. Patients were recruited from two wards of the same hospital, which simplified the process of adjusting the group.

Every translation of a health-related quality of life questionnaire requires a comprehensive, high-quality validation to provide the best adaptation to linguistic conditions [25]. This is a multi-stage and interdisciplinary process, requiring cooperation between the linguist, physician, and patient. While the overall circumstances, mental abilities, nuances of cultural background, diagnosis, and age group of the respondents are taken into consideration, proper customization is immensely elaborate. It is even more difficult in the case of children due to the limitations posed by their knowledge of the conceptual framework. All of the above may influence the level of understanding of the questions [26]. A thorough linguistic process gives a solid basis for the objectification of non-quantitative symptoms, facilitates the measurement of soft endpoints, and simplifies the thread of understanding between the pediatric patient and physician, resulting in treatment adjustment. A statistical analysis confirmed the usefulness of the tool. So far, the instrument has been translated to and validated in ten different languages [27]. It was proven to be useful in the assessment of HRQoL in patients with both organic and functional disorders [28,29].

Despite the accuracy and multiplicity of stages of the whole process, some limitations must be discussed. Our study has several limitations. This study was conducted at a single institution, which might have negatively impacted the respondents’ diversity despite the fact that the authors tried to diversify the respondent groups. Moreover, as the studied region is rather equal in terms of language, some questions can be understood differently in regions with a greater variety of dialects, for example, in Southern Poland. Nevertheless, Poland is a country where the language principles taught are unified, and the respondents were only native speakers of Polish. Apart from linguistic limitations, we must raise the topic of potential biases, especially recall bias and social desirability bias, connected to tabooed symptoms addressed in the PedsQL. For the group of younger children, patient–caregiver agreement bias is also probable. Unfortunately, these challenges are inherently difficult to eliminate entirely.

The utilization of questionnaires is particularly significant for the ongoing monitoring of chronic diseases, which are prevalent in pediatric gastroenterology (e.g., inflammatory bowel disease). These instruments not only facilitate informed decision-making regarding the optimization of therapeutic interventions but also contribute to the development of comprehensive, holistic care strategies, including psychological support or dietetic consulting. Furthermore, the assessment of disease severity enables the determination of appropriate timelines for follow-up consultations, directly influencing disease progression and enhancing patient well-being.

## 5. Conclusions

The final valid version of the questionnaire is ready to use. Linguistically, the questionnaire is complete. The high reliability of the questionnaire confirms its appropriateness for use in both clinical trials and routine clinical practice. Use of the version described here will allow for a more thorough assessment of the health status of a relatively large group of pediatric patients suffering from gastrointestinal tract ailments.

## Figures and Tables

**Table 1:** Analysis of comparisons for demographic variables in child and parent samples.

	Exposed	Control	*p*-Value
Children			
Count	100 (59.5)	68 (40.5)	-
Mean age of children	10.8 (10.0–11.6)	11.5 (10.6–12.4)	0.284
Child’s sex: male	44.0 (44.0)	35.0 (51.5)	0.427
Child’s sex: female	56.0 (56.0)	33.0 (48.5)	
Parent’s sex: male	16.0 (16.0)	5.0 (7.4)	0.154
Parent’s sex: female	84.0 (84.0)	63.0 (92.6)	
Education of parent: higher	45.0 (45.0)	35.0 (51.5)	0.708
Education of parent: secondary	34.0 (34.0)	22.0 (32.4)	
Education of parent: primary	1.0 (1.0)	0.0 (0.0)	
Education of parent: vocational	20.0 (20.0)	11.0 (16.2)	
Parents			
Count	125 (61.6)	78 (38.4)	-
Mean age of children	9.2 (8.4–10.1)	10.5 (9.5–11.5)	0.082
Child’s sex: male	56.0 (44.8)	42.0 (53.8)	0.267
Child’s sex: female	69.0 (55.2)	36.0 (46.2)	
Parent’s sex: male	18.0 (14.4)	5.0 (6.4)	0.129
Parent’s sex: female	107.0 (85.6)	73.0 (93.6)	
Education of parent: higher	59.0 (47.2)	40.0 (51.3)	0.791
Education of parent: secondary	42.0 (33.6)	26.0 (33.3)	
Education of parent: primary	1.0 (0.8)	0.0 (0.0)	
Education of parent: vocational	23.0 (18.4)	12.0 (15.4)	

**Table 2:** The results of the factor analysis.

Factor	Item	Factor Loading	AVE	CR
Stomach pain and hurt	I feel pain or hurt in my stomach	0.87 *	0.67	0.93
	I get stomach aches	0.87 *		
	My stomach hurts	0.82 *		
	I wake up at night with stomach aches	0.79 *		
	I feel sick to my stomach	0.70 *		
	I get an upset stomach	0.87 *		
Stomach discomfort when eating	When I eat I get sick to my stomach	0.64 *	0.63	0.89
	When I eat my stomach feels bad	0.84 *		
	My stomach hurts when I eat	0.80 *		
	My stomach feels heavy when I eat	0.87 *		
	I feel full as soon as I start to eat	0.79 *		
Food and drink limits	I cannot eat some foods	0.44 *	0.62	0.90
	I cannot drink some drinks	0.55 *		
	I am not able to eat what I want	0.62 *		
	I am not able to drink what I want	0.98 *		
	I cannot eat some foods because they make me sick	0.98 *		
	I cannot eat the foods that my friends eat	0.95 *		
Trouble swallowing	It is hard for me to swallow food	0.84 *	0.63	0.84
	It hurts when I swallow	0.79 *		
	Food gets stuck going down	0.76 *		
Heartburn and reflux	I get a burning feeling in my throat	0.53 *	0.66	0.88
	I have pain or hurt in my chest	0.95 *		
	I burp a lot	0.86 *		
	Food comes back up into my mouth after eating	0.85 *		
Nausea and vomiting	I feel like throwing up	0.77 *	0.48	0.78
	I feel like throwing up when I eat	0.62 *		
	I feel like throwing up after I eat	0.68 *		
	I throw up	0.69 *		
Gas and bloating	My stomach feels full of gas	0.73 *	0.67	0.93
	My stomach feels very full	0.84 *		
	My stomach gets big and hard	0.84 *		
	I have a lot of gas	0.90 *		
	I pass a lot of gas	0.75 *		
	My stomach feels gassy	0.79 *		
	My stomach makes noises	0.88 *		
Constipation	I still feel full after I poop	0.70 *	0.65	0.96
	I feel like I am not done after I poop	0.81 *		
	I feel like I cannot get all the poop to come out	0.84 *		
	It hurts when I go poop	0.84 *		
	My poop is hard	0.79 *		
	My poop is lumpy	0.78 *		
	I have to push hard to poop	0.87 *		
	My poop gets stuck when I poop	0.84 *		
	My bottom hurts after I go poop	0.74 *		
	It takes a long time for poop to come out	0.86 *		
	I have to work hard to make poop come out	0.87 *		
	I do not want to poop because it hurts	0.69 *		
	I spend a lot of time on the toilet going poop	0.82 *		
	My stomach hurts when I go poop	0.79 *		
Blood in bowel movement	There is blood on my toilet paper after I go poop	0.89 *	0.86	0.92
	There is blood in my poop	0.96 *		
Diarrhea	I need to be near the bathroom a lot	0.85 *	0.63	0.92
	I have to rush to the bathroom to poop	0.86 *		
	I feel like I am always in the bathroom going poop	0.82 *		
	I wake up at night to go poop	0.77 *		
	My poop is watery	0.77 *		
	I have poop accidents in my underwear	0.64 *		
	I have to go poop a lot	0.83 *		
Worry about bowel movements	I worry about going poop in my pants	0.91 *	0.58	0.87
	I worry that I will not make it to the bathroom in time	0.91 *		
	I worry that it will hurt when I go poop	0.57 *		
	I worry that I will have to use the bathroom at school	0.57 *		
	I worry that I will poop in my pants at school	0.77 *		
Worry about stomach aches	I worry about my stomach aches	0.94 *	0.90	0.95
	I worry that my stomach will hurt in school	0.96 *		
Medicine	It is hard for me to take my medicines	0.83 *	0.47	0.76
	I forget to take my medicines	0.36 *		
	It is hard for me to swallow my medicines	0.84 *		
	I do not like having to take my medicines all the time	0.61 *		
Communication	It is hard for me to tell the doctors and nurses how I feel	0.73 *	0.61	0.89
	It is hard for me to ask the doctors and nurses questions	0.69 *		
	It is hard for me to explain my illness to other people	0.96 *		
	It is hard for me to explain my illness to my friends	0.87 *		
	It is hard for me to talk to my parents about my illness	0.61 *		

Note. AVE—average variance extracted; CR—composite reliability; * *p* < 0.001.

**Table 3:** The results of the Fornell–Larcker criterion.

	1	2	3	4	5	6	7	8	9	10	11	12	13	14
1. Stomach pain and hurt	0.67													
2. Stomach discomfort when eating	0.38	0.63												
3. Food and drink limits	0.28	0.38	0.62											
4. Trouble swallowing	0.20	0.38	0.08	0.63										
5. Heartburn and reflux	0.26	0.63	0.33	0.34	0.66									
6. Nausea and vomiting	0.27	0.42	0.18	0.46	0.43	0.48								
7. Gas and bloating	0.22	0.40	0.36	0.16	0.36	0.24	0.67							
8. Constipation	0.23	0.28	0.24	0.21	0.17	0.22	0.42	0.65						
9. Blood in bowel movement	0.04	0.02	0.10	0.00	0.01	0.01	0.06	0.13	0.86					
10. Diarrhea	0.05	0.11	0.15	0.02	0.13	0.02	0.23	0.09	0.22	0.63				
11. Worry about bowel movements	0.04	0.04	0.09	0.02	0.04	0.01	0.19	0.17	0.18	0.56	0.58			
12. Worry about stomach aches	0.19	0.42	0.37	0.11	0.30	0.18	0.29	0.24	0.12	0.19	0.20	0.90		
13. Medicine	0.11	0.07	0.07	0.07	0.08	0.05	0.05	0.07	0.03	0.07	0.07	0.09	0.47	
14. Communication	0.18	0.22	0.19	0.14	0.17	0.11	0.09	0.17	0.04	0.05	0.09	0.26	0.17	0.61

Note. The AVE values are shown on the diagonal, with the bottom triangle being the squares of the covariance matrix.

**Table 4:** The results of the Mann–Whitney U test.

Dependent Variable	Children						*Z*	*p*	*r*
	Control Group (*n* = 68)			Exposed Group (*n* = 100)					
	Average Rank	Mdn	IQR	Average Rank	Mdn	IQR			
Stomach pain and hurt	52.40	5.50	8.00	106.33	0.00	2.00	−7.07	<0.001	0.55
Stomach discomfort when eating	54.42	8.00	13.00	104.96	0.00	3.75	−6.73	<0.001	0.52
Food and drink limits	56.03	0.00	2.00	103.86	0.00	0.00	−6.39	<0.001	0.49
Trouble swallowing	70.26	3.00	6.00	94.19	0.50	2.00	−3.66	<0.001	0.28
Heartburn and reflux	66.12	4.50	7.00	97.00	0.00	2.75	−4.16	<0.001	0.32
Nausea and vomiting	57.66	12.00	12.50	102.75	3.00	7.75	−6.06	<0.001	0.47
Gas and bloating	54.60	17.50	25.50	104.84	4.00	11.00	−6.58	<0.001	0.51
Constipation	55.68	0.00	3.00	104.10	0.00	0.00	−6.35	<0.001	0.49
Blood in bowel movement	69.34	5.50	9.00	94.81	1.00	3.00	−4.28	<0.001	0.33
Diarrhea	59.62	4.00	9.75	101.42	0.50	4.00	−5.53	<0.001	0.43
Worry about bowel movements	64.32	4.00	5.75	98.22	0.00	2.00	−4.54	<0.001	0.35
Worry about stomach aches	59.42	3.50	6.00	101.56	0.00	2.00	−5.69	<0.001	0.44
Medicine	64.60	8.00	9.00	98.03	1.00	4.75	−4.53	<0.001	0.35
Communication	59.38	0.00	0.00	101.58	0.00	0.00	−5.61	<0.001	0.43
	Parents								
	Control group (*n* = 78)			Exposed group (*n* = 125)					
Stomach pain and hurt	59.40	0.00	1.00	128.58	5.00	8.50	−8.18	<0.001	0.57
Stomach discomfort when eating	64.62	0.00	2.00	125.32	7.00	12.00	−7.49	<0.001	0.53
Food and drink limits	68.76	0.00	0.00	122.74	0.00	2.00	−6.51	<0.001	0.46
Trouble swallowing	84.87	0.00	1.00	112.69	2.00	6.00	−3.93	<0.001	0.28
Heartburn and reflux	73.26	0.00	1.00	119.94	3.00	6.50	−5.76	<0.001	0.40
Nausea and vomiting	71.28	1.00	4.25	121.17	14.00	12.00	−6.13	<0.001	0.43
Gas and bloating	58.27	3.00	7.00	129.29	20.00	23.50	−8.41	<0.001	0.59
Constipation	62.37	0.00	0.00	126.73	0.00	2.00	−7.61	<0.001	0.53
Blood in bowel movement	80.01	0.00	2.00	115.72	5.00	9.00	−5.39	<0.001	0.38
Diarrhea	67.25	0.00	2.00	123.68	4.00	7.00	−6.78	<0.001	0.48
Worry about bowel movements	73.57	0.00	1.00	119.74	3.00	5.50	−5.62	<0.001	0.39
Worry about stomach aches	70.06	0.00	2.00	121.93	3.00	6.00	−6.47	<0.001	0.45
Medicine	80.22	0.00	4.25	115.59	7.00	9.00	−4.42	<0.001	0.31
Communication	71.17	0.00	0.00	121.24	0.00	0.00	−6.04	<0.001	0.42

Note. *n*—number of observations; Mdn—median; IQR—interquartile range; *Z*—value of test statistic; *p*—statistical significance; *r*—strength of effect index.

**Table 5:** An analysis of the convergence of children’s and parents’ responses.

	Parents	
Children	Spearman’s *rho*	*p*
Stomach pain and hurt	0.88	<0.001
Stomach discomfort when eating	0.80	<0.001
Food and drink limits	0.78	<0.001
Trouble swallowing	0.68	<0.001
Heartburn and reflux	0.70	<0.001
Nausea and vomiting	0.87	<0.001
Gas and bloating	0.79	<0.001
Constipation	0.89	<0.001
Blood in bowel movement	0.84	<0.001
Diarrhea	0.82	<0.001
Worry about bowel movements	0.79	<0.001
Worry about stomach aches	0.72	<0.001
Medicine	0.69	<0.001
Communication	0.74	<0.001

**Table 6:** The results of the discriminant analysis.

Variable		Children		Parents	
		Control Group (*n* = 68)	Exposed Group (*n* = 100)	Control Group (*n* = 78)	Exposed Group (*n* = 125)
Stomach pain and hurt		0.18	0.27	0.19	0.25
Stomach discomfort when eating		−0.12	−0.05	−0.11	−0.01
Food and drink limits		−0.02	0.07	0.00	0.07
Trouble swallowing		−0.06	−0.15	0.00	−0.17
Heartburn and reflux		0.12	0.14	0.02	0.22
Nausea and vomiting		−0.11	−0.10	−0.06	−0.22
Gas and bloating		0.10	0.13	0.05	0.21
Constipation		0.00	0.03	0.01	0.05
Blood in bowel movement		−0.15	0.07	−0.11	0.13
Diarrhea		0.03	0.14	0.03	0.18
Worry about bowel movements		0.01	−0.05	−0.01	−0.20
Worry about stomach aches		−0.07	−0.19	−0.04	0.08
Medicine		0.11	0.16	0.10	0.16
Communication		0.07	0.19	0.08	0.24
Constant		−1.38	−4.46	−1.30	−5.41
Classification results	Control group	60 (88.2%)	8 (11.8%)	70 (89.7%)	8 (10.3%)
	Criterion group	22 (22.0%)	78 (78.0%)	21 (16.8%)	104 (83.2%)

Note. Loads were calculated based on Fisher’s discriminant linear functions.

**Table 7:** Cronbach’s alpha in exposed vs. control group (child and parent populations).

Cronbach’s Alpha	Children		Parents	
	Exposed	Control	Exposed	Control
Stomach pain and hurt	0.859 (0.853–0.864)	0.89 (0.887–0.893)	0.847 (0.841–0.853)	0.89 (0.886–0.895)
Stomach discomfort when eating	0.834 (0.819–0.849)	0.715 (0.701–0.73)	0.854 (0.844–0.865)	0.544 (0.459–0.63)
Food and drink limits	0.879 (0.865–0.893)	0.821 (0.809–0.833)	0.929 (0.925–0.933)	0.792 (0.748–0.836)
Trouble swallowing	0.802 (0.791–0.812)	0.354 (0.268–0.441)	0.831 (0.808–0.855)	0.526 (0.466–0.587)
Heartburn and reflux	0.748 (0.731–0.765)	0.563 (0.557–0.57)	0.78 (0.768–0.792)	0.34 (0.21–0.471)
Nausea and vomiting	0.845 (0.832–0.859)	0.801 (0.794–0.808)	0.863 (0.85–0.876)	0.864 (0.855–0.873)
Gas and bloating	0.891 (0.884–0.898)	0.868 (0.862–0.873)	0.918 (0.915–0.922)	0.919 (0.912–0.926)
Constipation	0.947 (0.942–0.953)	0.932 (0.928–0.936)	0.954 (0.952–0.956)	0.937 (0.932–0.943)
Blood in bowel movement	0.901 (0.878–0.924)	0.333 (0.191–0.474)	0.897 (0.881–0.914)	0.070 (0.010–0.120)
Diarrhea	0.911 (0.908–0.915)	0.718 (0.678–0.758)	0.917 (0.913–0.921)	0.807 (0.793–0.821)
Worry about bowel movements	0.852 (0.848–0.857)	0.786 (0.758–0.813)	0.854 (0.844–0.865)	0.717 (0.704–0.729)
Worry about stomach aches	0.959 (0.956–0.961)	0.892 (0.885–0.9)	0.902 (0.891–0.913)	0.773 (0.703–0.844)
Medicine	0.649 (0.63–0.668)	0.676 (0.648–0.705)	0.718 (0.705–0.732)	0.848 (0.835–0.861)
Communication	0.876 (0.869–0.884)	0.783 (0.748–0.817)	0.898 (0.889–0.906)	0.832 (0.811–0.853)
All questions	0.946 (0.94–0.952)	0.951 (0.947–0.955)	0.931 (0.925–0.937)	0.953 (0.95–0.956)

## Data Availability

The data are available from the corresponding author upon reasonable request.

## Appendix A

**Table A1:** Distributions of test items for full study sample (*n* = 371).

Item	Skewness	Kurtosis
I feel pain or hurt in my stomach	0.03	−0.97
I get stomach aches	0.02	−1.03
My stomach hurts	0.07	−0.97
I wake up at night with stomach aches	1.38	0.96
I feel sick to my stomach	0.78	−0.57
I get an upset stomach	1.13	0.35
When I eat I get sick to my stomach	1.20	0.17
When I eat my stomach feels bad	1.25	0.69
My stomach hurts when I eat	1.36	0.79
My stomach feels heavy when I eat	1.30	0.64
I feel full as soon as I start to eat	1.10	−0.01
I cannot eat some foods	0.60	−1.00
I cannot drink some drinks	0.90	−0.40
I am not able to eat what I want	0.98	−0.29
I am not able to drink what I want	1.35	0.64
I cannot eat some foods because they make me sick	1.33	0.59
I cannot eat the foods that my friends eat	0.90	−0.64
It is hard for me to swallow food	2.16	3.79
It hurts when I swallow	2.70	6.72
Food gets stuck going down	2.45	5.61
I get a burning feeling in my throat	1.99	3.52
I have pain or hurt in my chest	1.64	1.58
I burp a lot	1.05	0.07
Food comes back up into my mouth after eating	1.94	2.95
I feel like throwing up	0.71	−0.55
I feel like throwing up when I eat	1.60	1.77
I feel like throwing up after I eat	1.20	0.33
I throw up	1.45	0.97
My stomach feels full of gas	0.56	−0.93
My stomach feels very full	0.44	−1.07
My stomach gets big and hard	0.74	−0.71
I have a lot of gas	0.45	−1.07
I pass a lot of gas	0.48	−1.05
My stomach feels gassy	0.61	−0.81
My stomach makes noises	0.41	−0.90
I still feel full after I poop	1.13	0.10
I feel like I am not done after I poop	0.76	−0.60
I feel like I cannot get all the poop to come out	0.63	−0.78
It hurts when I go poop	0.85	−0.41
My poop is hard	0.66	−0.67
My poop is lumpy	0.94	−0.23
I have to push hard to poop	0.71	−0.70
My poop gets stuck when I poop	1.08	−0.11
My bottom hurts after I go poop	1.21	0.37
It takes a long time for poop to come out	0.68	−0.78
I have to work hard to make poop come out	0.79	−0.72
I do not want to poop because it hurts	1.65	1.63
I spend a lot of time on the toilet going poop	0.75	−0.71
My stomach hurts when I go poop	1.00	−0.27
There is blood on my toilet paper after I go poop	1.96	2.73
There is blood in my poop	2.17	3.56
I need to be near the bathroom a lot	1.41	0.99
I have to rush to the bathroom to poop	1.22	0.41
I feel like I am always in the bathroom going poop	1.75	1.97
I wake up at night to go poop	1.81	2.40
My poop is watery	1.06	−0.06
I have poop accidents in my underwear	1.13	0.15
I have to go poop a lot	1.39	1.16
I worry about going poop in my pants	1.53	1.23
I worry that I will not make it to the bathroom in time	1.42	1.16
I worry that it will hurt when I go poop	1.34	0.70
I worry that I will have to use the bathroom at school	1.01	−0.23
I worry that I will poop in my pants at school	1.90	2.68
I worry about my stomach aches	0.66	−0.97
I worry that my stomach will hurt in school	0.74	−0.74
It is hard for me to take my medicines	1.60	1.58
I forget to take my medicines	1.49	1.50
It is hard for me to swallow my medicines	1.38	1.04
I do not like having to take my medicines all the time	1.21	0.06
It is hard for me to tell the doctors and nurses how I feel	0.87	−0.48
It is hard for me to ask the doctors and nurses questions	0.85	−0.51
It is hard for me to explain my illness to other people	0.62	−0.88
It is hard for me to explain my illness to my friends	0.65	−0.93
It is hard for me to talk to my parents about my illness	1.63	1.73

## Appendix B

**Table A2:** Distribution of tested variables in each group.

Variable	Children						Parents					
	Control Group (*n* = 68)			Exposed Group (*n* = 100)			Control Group (*n* = 78)			Exposed Group (*n* = 125)		
	*As*	*K*	*p*	*As*	*K*	*p*	*As*	*K*	*p*	*As*	*K*	*p*
Stomach pain and hurt	1.18	0.76	<0.001	−0.16	−0.77	0.03	1.32	1.66	<0.001	0.08	−0.69	<0.001
Stomach discomfort when eating	1.64	2.27	<0.001	0.54	−0.66	<0.001	2.99	10.16	<0.001	0.66	−0.24	<0.001
Food and drink limits	1.73	2.29	<0.001	0.30	−1.13	<0.001	2.04	3.21	<0.001	0.51	−0.81	<0.001
Trouble swallowing	2.67	6.97	<0.001	1.56	1.31	<0.001	2.78	7.16	<0.001	2.11	3.88	<0.001
Heartburn and reflux	1.43	1.51	<0.001	0.89	0.13	<0.001	3.57	17.08	<0.001	1.30	1.46	<0.001
Nausea and vomiting	1.56	1.84	<0.001	0.57	−0.30	<0.001	2.82	8.82	<0.001	0.88	0.13	<0.001
Gas and bloating	1.21	1.11	<0.001	0.22	−0.95	<0.001	1.89	3.53	<0.001	−0.22	−0.93	<0.001
Constipation	1.94	4.56	<0.001	0.57	−0.66	<0.001	2.72	9.77	<0.001	0.31	−0.83	<0.001
Blood in bowel movement	2.96	7.29	<0.001	1.39	0.69	<0.001	4.11	16.14	<0.001	1.46	0.88	<0.001
Diarrhea	1.91	3.45	<0.001	0.95	0.08	<0.001	2.43	6.03	<0.001	1.09	0.48	<0.001
Worry about bowel movements	2.07	5.56	<0.001	0.73	−0.48	<0.001	1.91	3.34	<0.001	1.04	0.24	<0.001
Worry about stomach aches	1.71	2.24	<0.001	0.01	−1.28	<0.001	2.31	5.13	<0.001	0.32	−1.15	<0.001
Medicine	2.52	8.10	<0.001	0.73	0.23	<0.001	2.27	5.47	<0.001	0.50	−0.97	<0.001
Communication	1.75	3.32	<0.001	0.24	−0.82	<0.001	1.82	3.57	<0.001	0.44	−0.72	<0.001

Note. *n*—number of observations; *As*—skewness; *K*—kurtosis; *p*—significance of Shapiro–Wilk test.
